# Genetics and genomic medicine in Morocco: the present hope can make the future bright

**DOI:** 10.1002/mgg3.255

**Published:** 2016-11-10

**Authors:** Khadija Belhassan, Karim Ouldim, Abdel Aziz Sefiani

**Affiliations:** ^1^ Medical Genetics and Onco‐Genetics DepartmentHassan II University Medical CenterFezMorocco; ^2^ Medical Genetics DepartmentNational Institute of HygieneRabatMorocco

## Abstract

Genetics and genomic medicine in Morocco: the present hope can make the future bright.

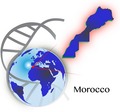

## Introduction

The status of medical genetics and genomics medicine in Morocco cannot be truly understood without considering the general health system components in the country, policy making, social factors, cultural behaviors, and even historical events, within the geographic state. It is important to note that medical genetics does not operate in a vacuum, but with all of these factors in a symbiotic relationship.

## General Statistics, Geography, History, and Political Overview

**Figure 1:**
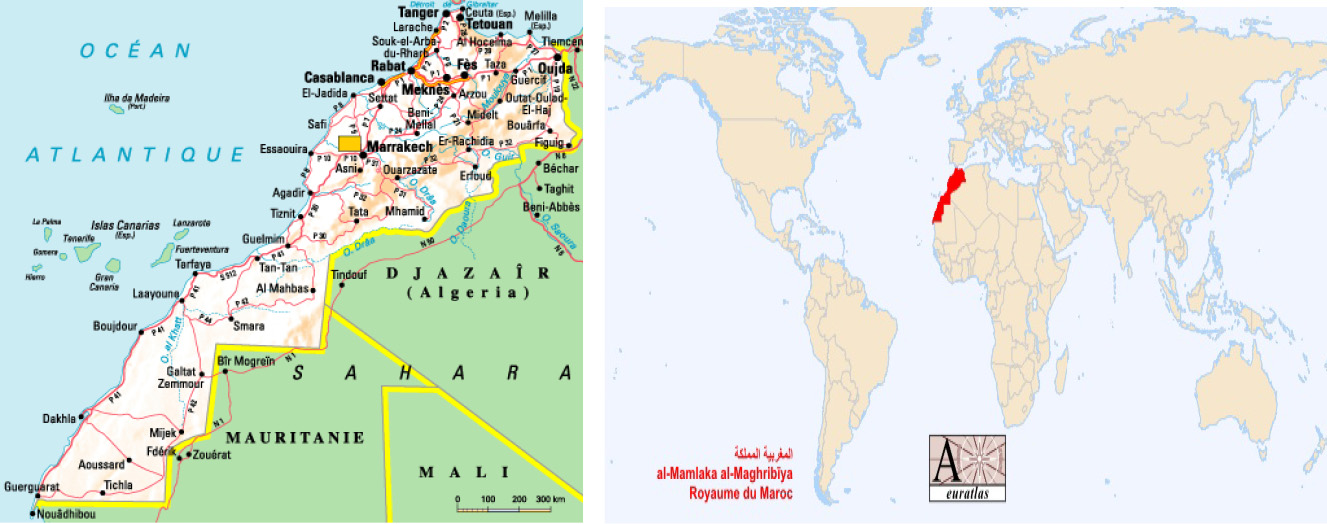
Morocco Territory Map, with its geographic opening to the European continent.

The kingdom of Morocco occupies 710.850 square kilometers, spanning from the Mediterranean Sea and Atlantic Ocean on the north and the west, respectively, into the borders of Algeria and Mauritania on the East and south. A large mountainous area occupies the interior body of the country, with a desert area in the far south. Morocco is located in the extreme northwest of Africa at the doors of continental Europe. The Mediterranean Strait of Gibraltar opens Moroccan territories to the European continent via Spain (Charoute et al. 2014; Geography of Morocco, 2016) (Figure 1).

The Moroccan Monarchy called “Allawi Monarchy” has reigned over Morocco since the 17th century. The kingdom became a French protectorate in 1912, and gained independence in 1956. Since its independence, Moroccan domestic and foreign policies have focused on maintaining Morocco unity and working toward peace in the Middle East. (James 2016; The World of Royalty, 2016).

As of 2014, the population of Morocco was estimated to be 33,848,242. About 58% of the population is urban. (royaume du Maroc, 2016). Urban migration has been steadily increasing over the last two decades, increasing from 51.5% to 55% between 1994 and 2004 and then to 58% in 2014. This phenomenon presents a serious problem for policy planners due to the heavy stress it places on services in major cities (Morocco Health Ministry, 2013).

Morocco's birth rate stood at 18.8 per 1.000, whereas the death rate was reported at 5.6 per 1.000, with a projected annual growth rate of 1.09%. The population up to age 60 years is expected to reach a percentage of 11.5% by the year 2020, while the population less than 15 years will reach 20.9% in 2030.

The vast majority of the population is Muslim; the other small religious groups represent Jews and Christians. The ethnic population diversity includes mainly Arabs, Berbers, and Hassani (Sahrawi), with some individuals of Phoenicians, Romans, Vandals, Byzantines, Moriscos, sub‐Saharian Africans, and European remnants of the colonial period (Ratbi et al. 2008; Morocco Health Ministry, 2013).

Morocco is a country of immigrants: nearly 1.8 million Moroccans live abroad, in particular in France, Italy, Spain, and Belgium (Ratbi et al. 2008).

Analphabetism decreased from 55% to 43% from 1994 to 2004.

Morocco's general health policy focuses on the promotion of primary health care, encouragement of family planning especially in rural areas, and the reduction in infant and maternal mortality (Morocco Health Ministry, 2013).

## Economy, Education Policies

The agricultural sector contributes 19% to the national GDP, 15% in agriculture and 4% in agribusiness. This sector employs over 4 million rural persons and creates about 100,000 jobs in the field of food. The income of 80% of the rural populations depends on agriculture. Agriculture accounts for between 14 and 24% of total imports and between 15 and 21% of total exports (Moroccan National Gate, 2016).

The long coastline of 2945 km length makes the fisheries sector an important part of the economy. Other natural resources include wood and minerals (phosphates, iron, manganese, lead, and zinc) (Moroccan National Gate, 2016).

Morocco has identified tourism as a priority sector in its development strategy. (Moroccan National Gate, 2016) This Tourism program, developed jointly by private sector operators and government, has concentrated on road network development, placing Morocco among the first African countries to have such infrastructure. Sixty per cent of its 60.000 km road network is surfaced, whereas 1500 km of roads are built every year. In addition, the Mediterranean by‐pass is under construction (Moroccan National Gate, 2016).

Morocco continues to stress the importance of education in social development and economic growth. The government advocates for free basic education especially in rural areas with a promotion of girls' education. (Moroccan National Gate, 2016) 61.5% of the population over the age of 10 years is without any formal educational qualifications, while 30.5% have either a primary or fundamental school certificate. Only 8% have a high school degree education or more (Morocco Health Ministry, 2013).

From a social standpoint, nearly 5 million Moroccans live in substandard housing. This situation does not only distort and disfigure the Moroccan urban landscape, but also it is the bed of the proliferation of many social illnesses. A project, spearheaded by The National Initiative for Human Development, has been undertaken to eradicate slums, to provide adequate housing for individuals in proportion to their resources and to provide economic opportunities (Moroccan National Gate, 2016).

## Health Care System in Morocco

According to the World Health Organization, the health system includes all activities, formal or informal, which focuses on health services available to a population to meet their needs in terms of health. The role of any health system is to first identify the needs of its population and to secondly implement the policies and actions that can meet that needs (World Health Organization about Morocco, 2016).

The Morocco health care system is based upon the principles of solidarity and social cohesion. The system is characterized by universal health insurance for Moroccans, with public financing of primary health care, and an orientation care network focused on population health needs. This system is structured by levels with territorial and regional organization, a public/private partnership, an intersector collaboration, and a specialization of general practitioners in the primary health care (Morocco Health Ministry, 2016).

A compulsory health coverage system (AMO) and regime of health assistance (RAMED) (for the benefit of deprived persons based on the principles of social cohesion and national solidarity) were, respectively, created in 2005 and 2011, to provide universal access to health care. Both systems cover approximately 60% of the population (Morocco Health Ministry, 2016).

The health global spending reached approximately 47.8 billion Dirhams in 2010, or 1498 Dirhams per capita (181 US dollars in 2010). These expenses represent 6.2% of the Gross Domestic Product, compared to 5.3% in 2006 (Morocco Health Ministry, 2013).

For a population of about 33 million, the number of physicians is 19.770, whereas nurses account for 29.025 (Morocco Health Ministry, 2013).

The number of urban basic health care facilities is 2689, whereas the number of rural basic health care facilities is 1938, providing a basic health care center per 12.000 habitants (Morocco Health Ministry, 2013).

There are 33 local hospital, 25 regional hospital centers, and five university hospital centers, with two others under constructions. (Morocco Health Ministry, 2013).

There are four specialized laboratory centers for dealing with tuberculosis, family planning, diagnostics, and cancer screening. (Morocco Health Ministry, 2013).

Chronic diseases accounts for 18% affecting mainly females and elders, thus, the health system priorities are fighting chronic diseases such as diabetes and arterial hypertension. The system aims also to prevent infectious diseases by immunization program implementation, and treating sexually transmitted infections (Morocco Health Ministry, 2013).

Other priority areas are providing free cancer treatment, especially for breast and cervical cancer, and reducing infant mortality. In Morocco, out of every 1000 liveborn children, 29 infants die before reaching one year, 19 during the first month of life and 10 between the 1^st^ and 12th months. Thus, in 1000 infants aged one year, two do not reach their 5th year. The probability a child dies before reaching 5 years is 30 for 1000. To address this issue, there are programs to reduce maternal mortality rates by providing free childbirth delivery, to encourage breast feeding, and to provide adequate nutritional intake; 3.1% of children are underweight (Morocco Health Ministry, 2013).

The Moroccan national household mean size is 4.9. Fecundity has diminished to 2.5 children due to contraception and marriage age increase. 56.6% of the population up to 15 years is married.

Access to water, electricity and sanitation is also a priority for the health system (Morocco Health Ministry, 2013).

## Medical Genetics in Morocco, a Population Marked by High Consanguineous Marriage Rate

Medical genetics is a new specialty in Morocco, recognized since 1996. Thus, Morocco is among the first countries to have recognized this specialty, just after France since 1995.

The pioneer and first medical geneticist in the country is Dr. Abdel Aziz Sefiani, head of the Department of Medical Genetics in Rabat National Institute of Hygiene. Since then, other departments have been established. A Department of Genetics in Casablanca faculty of medicine was established followed by another one in Fes Hassan II University medical center. Less than 30 Medical genetics practitioners and trainees are enrolled in the field. The specialty is blossoming in five university medical centers; more medical geneticists will turn to this specialty which is a mixture of medical practice and research. In addition two international university hospitals in Casablanca and Rabat, with both public/private partners are adopting medical genetics in their facility. Ideally, Moroccan geneticists will establish university hospital medical genetics units with reference centers in the various domains of medical genetics. To accomplish this, it is necessary to increase the number of medical geneticists in Morocco.

### Consanguinity

Like many North African or Middle Eastern groups where the consanguinity rate is increased, Morocco consanguineous marriages account for 19.9–28% of all marriages, with 50% of these between first cousins. (Tadmouri et al. 2009) A recent study estimates the frequency of consanguineous marriages to be 59.09% among families with autosomal recessive disorders (Jaouad et al. 2009). Despite this high rate of consanguinity, information on inherited disorders in Morocco is not extensive. There are only few detailed studies on the prevalence of some diseases such as familial Mediterranean fever, cystic fibrosis, and beta thalassemia. Nevertheless, an important sporadic data are available for more than 250 genetic diseases and syndromes (Ratibi et al. 2008; Charoute et al. 2014). Despite the lack of genetic diseases epidemiological surveillance, they occupy a more and more important sphere in morbidity, but they stimulate only a humble interest in Moroccan health care system strategy. Birth defects, inborn errors of metabolism, mitochondrial diseases, primary immunodeficiency, hereditary diseases, and cancers affect thousands of children and adults, and are a source of both economic and psychological burden for families. Raising awareness of these disorders is a key need.

### Diagnostic tests

The Moroccan newsletter “Libération” reported in 1993 on the first molecular genetic test performed in the Moroccan National Institute of Hygiene to diagnose Duchenne Muscular Dystrophy. It was an ambitious, optimistic step for this new field of Medical Genetics and molecular biology in the country. Since then, several genetic tests have been developed and patients with common genetic or inherited diseases in Morocco can benefit from genetic diagnosis (Table 1).

**Table 1:** Twenty years of cytogenetics molecular cytogenetics and molecular biology in Morocco

Disease	Diagnosis method
Constitutional chromosomal abnormalities	Cytogenetic and molecular cytogenetics
Williams syndrome	Cytogenetic and molecular cytogenetics (FISH)
Digeorge syndrome	Cytogenetic and molecular cytogenetics (FISH)
Prader Willi and Angelman syndromes	Cytogenetics molecular cytogenetics and molecular biology (methylation status of 15q11.2)
Chronic myeloid leukemia	Cytogenetic and molecular cytogenetics
Chronic myeloid leukemia	Molecular biology of (BCR/ABL: by RT‐PCR)
Myeloproliferative syndromes	Molecular biology (V617F mutation in JAK2 gene)
Myelofibrosis with myeloid metaplasia, essential thrombocythemia	Molecular biology of (Exon 9 of CALR gene)
Familial Mediterranean fever	Molecular biology (MEFV gene)
Autosomal recessive Limb‐Girdle muscular dystrophy type 2C	Molecular biology (SGCG gene/525 delT mutation)
Muscular dystrophy Duchenne and Becker type	Molecular biology
Spinal muscular atrophy	Molecular biology
Deafness due to connexin 26 anomalies	Molecular biology of (GJB2 gene/35delG mutation)
Factor V Leiden mutation	Molecular biology
Beta‐thalassemia and hbb‐related diseases	Molecular biology of (Moroccan recurrent mutations)
Cystic fibrosis	Molecular biology of (Exon 10 of CFTR gene)
Attenuated familial adenomatous polyposis	Molecular biology of (MYH gene/Moroccan recurrent Mutations)
Mucopolysaccharidosis type 1	Molecular biology of (IDUA gene/c.3233C>G mutation)
Glycogen storage disease type IA	Molecular biology of (Moroccan recurrent mutations)
Xeroderma pigmentosum	Molecular biology of (XPC gene/c.1643_1644delTG mutation)
Hyperoxaluria	Molecular biology of (AGXT gene/p.Ile244Thr mutation)
Achondroplasia and hypochondroplasia	Molecular biology of (FGFR3 gene)
Hemochromatosis	Molecular biology of (HFE gene: C187G and G845A mutations)
Male infertility	Molecular biology of Y chromosome deletions (AZF)
Nephronophtisis	Molecular biology of (recurrent deletion of NPHP1 gene)
Familial hypercholesterolemia	Molecular biology (Moroccan recurrent mutations)
Pharmacogenetics	Molecular biology analysis of IL28B gene
Molecular diagnosis of male infertility associated with large‐headed multiflagellar polyploid spermatozoa	Molecular biology (AURKC gene: c.144delC mutation)

It is important for families and physicians to realize the importance of a diagnosis, even if the child dies. A diagnosis will allow more appropriate genetic counseling where recurrence rates can be discussed. Some genetic diseases are well known, identified well and easy to diagnose by the practitioners on the basis of their clinical expertise and without resorting to expensive equipment. Others will require the support of specialized examinations or imaging, or genetic biochemical analyses to end up with a precise diagnosis. Genetic diseases are not registered within the framework of the Moroccan health policy; they are, however, recognized as a problem of public health by specialists working with many patients with genetic illness.

With all the challenges facing the medical genetics field in Morocco, the first genetics health care professionals in the country were been promoting the field and trying to offer services to a population neglected by the health system. The limited resources, especially in an era of new generation sequencing, leave many genetic conditions undiagnosed, including those contracted in daily routine practice such as: **Autism**, although no exact statistics about the prevalence of the disorder in Morocco exist, support groups were created to allow the parents of autistic children to meet each other in order to exchange experiences and create awareness about autism. **Type1 diabetes**, where the genetic predisposition is elucidating more about the disease and should consequently be taken into consideration by the country health program. **Hemophilia**, According to the World Health Organization, more than 3000 people suffer from hemophilia in Morocco. Unfortunately, this number still is too low for the disease to be a priority of the Moroccan health system. Nevertheless, without adapted management, the hemophilia quickly becomes a handicap and a real ordeal for whole family. The primary preventive strategy represents the best alternative. In Morocco, the cost of antihemophilic factors is particularly costly and cannot be afforded by most families. Only in case of emergency, treatment can be delivered. **Primary Immunodeficiency**, Morocco is sorely lacking units and health care professionals specialized in management of children suffering from primary immunodeficiency. There are only two medical professional specialized in this field in the whole country, a very insufficient number with regard to the increasing number of children suffering from PID. An adequate bone marrow transplantation platform is absent in the country. Every month the health care system witnesses powerlessly the agony of one to two children because that system does not have the means to handle their illnesses. While in other countries such as Tunisia and Saudi Arabia, the children suffering from these pathologies can receive a transplant and live a normal life. It is urgent to react to improve an intolerable situation by setting up quickly units specialized to improve significantly the quality of life of these children. **Sexual Differentiation Disorders**, hundreds of cases are encountered in the Moroccan health facilities. This figure is, however, far from reflecting the reality because most of the parents and people affected by these disorders do not consult the specialized centers. Many parents refuse categorically the idea that their boys, raised as boys since their birth, are in fact girls. For them, it is a real drama to see their child changing sex specially that they do not consult at an early stage of the disorder. The decision of a surgical operation should be taken in dialog with the doctors of various specialties who estimate, in the light of the biological, anatomical, genetic, and psychological data which they have, the chances of success of such intervention, then with the parents of the child. This approach allows minimizing the risks related to the impact of sex reparation on the child. Some individuals have attempted suicide because they were conflicted over their new identity. Pediatricians and the general practitioners have an important role in the diagnosis of the disorders and the orientation of the children and the parents toward the specialized centers. **Congenital skeletal malformations**, many skeletal malformations are genetic or inherited. Prenatal diagnosis can be made by the ultrasound. The shock of the announcement of a deformation, the possibilities of management of this deformation explain the importance of the prenatal consultations and genetic counseling. **Congenital heart defects**, with a rough frequency of 8 for 1000 births, constitutes one of the first causes of infant mortality in Morocco, and remains a considerable cause of handicap of the child and the young adult. Although the cause may remain unknown in many cases, genetic causes, in particular chromosomal aberrations such as trisomy 21, will account for a significant number. Prenatal diagnosis represents an essential aspect in preventing cardiac birth defects. Heart surgery requires an adapted infrastructure, a multidisciplinary team and the development of specialized surgical skills. Management of congenital heart diseases thus represents a heavy cost, out of reach for the majority of the patients still not benefiting from health coverage (Doctinews, 2016).

As awareness of genetic disorders has increased both within the general population and among health care providers, interest in genetics has increased. Many genetic diseases are responsible for a very high mortality rate, and the high infant mortality can be explained partly due to undiagnosed genetic disease. The media in Morocco has tried to disseminate genetic information about lysosomal storage diseases, glycogen storage diseases, and inborn errors of metabolism in a country that lacks enzyme replacement therapy and reliable centers for bone marrow engraftment. Until recently, the Morocco ministry health system did not adopt strategies specific to control genetic conditions; many patients affected by these diseases are managed by cooperative medical societies and nongovernmental associations (Table 2).

**Table 2:** Nongovernmental institutions offering services for patients with genetic conditions

Association	Targeted population
Moroccan society of medical genetics	Population with genetic conditions
Moroccan society of clinical chemistry and medical biology(SMCC‐BM)	Population with metabolic conditions
Mirror association for autistic child	Population with autism
Hemophilic Moroccan association	Population with hemophilia
Moroccan association of autoimmune and systemic diseases (AMMAIS)	Population with autoimmune and systemic diseases
Overcome autism association	Population with autism
Pediatrics Moroccan association	Children including those with primary immune deficiency
Pediatric ORL Moroccan association	Population including congenital deafness
Moroccan association against myopathy (AMM)	Population with myopathies

## New Born Screening and Genetic Counseling

According to the international projections, the number of newborns with a genetic condition in Morocco will be 5% (Dr Anwar Cherkaoui, 2001).

Management of genetic conditions can be laborious and expensive. Some of these diseases are chronic and require therapeutic strategies which can cost up to 100 000 Dirhams a month. The costs are too much to be covered by health insurance, much less most families. Until now, families are left to their own destiny. The situation is even worse for families with multiple affected members. When will Morocco take care of these patients? A prevention policy costs less than a management one, but, Where? How? Who? When?

In this context, it is clear that Morocco with its national health policies should quickly start to integrate a strategy of prevention and raising awareness that genetic diseases are a serious health issue. The systematic screening of certain genetic diseases is not practiced in Morocco, but some pilot studies have been conducted, in particular for congenital hypothyroidism. The problem of the systematic screening is not the screening in itself rather the necessary logistics and the budget for a real national program which would support it. Newborn screening requires a great infrastructure to ensure collection of appropriate samples from all newborns that are then forwarded to reference laboratories, recontact of infants that screen positive, arranging diagnostic testing, and then management of the positive cases.

Ethically, it is not reasonable to screen for certain genetic diseases while being unable to treat or effectively manage already diagnosed patients. A targeted screening and prevention strategy toward high‐risk families at risk to have another affected child can be adopted to avoid this possible fact.

The diagnosis of a genetic disease sometimes requires expensive examinations tools, such as Exome Sequencing. Thus, certain genetic diagnostic tools are expensive, or even not available in Morocco, especially enzymology examinations and NGS examinations, but there is tendency to forget the cost of the undiagnosed such as: hospitalizations expenses, and families' useless unlimited travel expenses, which could be avoided in many cases. (Morocco genetic team personal experience).

### Genetic counseling

Given all the boundaries cited previously about the high morbidity of genetic diseases, the lack of understanding of genetics and genetic disease in both the general population, and the medical profession, health system priorities which exclude genetic diseases from their medical strategies, the country economic limitation resources, and the heavy burden of genetic disease diagnosis and management, genetic counseling can be an important tool for families. Genetic counseling is particularly important in Morocco when considering the high rate of consanguineous matings. A prophetic Islamic religious text says: *“Choose well your mate (for your gamete) as (the hidden) traits can reappear.”*

Some affected families may believe they got the illness as a punishment from God, others consider it as God's test or message, and they react differently dealing with the situation, by different mature and immature self‐ego defense mechanisms. They tend to condemn the opposite side, to fear stigmatization, or to reject the whole situation by putting the affected child in an orphan center.

Genetic counseling can help individuals understand inheritance patterns and the risk of genetic disease in consanguineous marriages. We have seen many families with three or four affected children and who declare they would not have had additional children if they had been warned from the first birth about a risk related to consanguinity. Thus genetic counseling is not only about assessing medical risk, it is about using psychology and knowledge about social and cultural issues to empower families to help them make the most appropriate decisions for their family. The low literacy in Morocco may foster a low understanding of genetic concepts, but, on the other hand, geneticists have poor skills simplifying the material as to be received easily by patients. This is due to the fact that in Morocco, there is no specialization in genetic counseling, geneticist have to be the genetic counselor often with no psychological skills background. Another big issue involves the unavailability of formal legislation regulating the practice of genetics, and unavailability of alternative choices for families, either by preimplantation genetic diagnosis, or prenatal genetic diagnosis (Morocco medical genetics team personal experience).

## Medical Genetics Legislations and Reproductive Law

With the fast continuous advances in genetics and genomic technologies, ethical issues emerge, and bring to the surface the immense need to set up regulations which would frame and supervise the practice of genetics in Morocco. Since the government cannot afford financially all aspect of genetics, it has at least the obligation to set up regulations for the private sector that are investing in the field. Regulations have to include genetic test charges and ensuring that testing laboratories have necessary practice approvals, have a trained staff and are awarded a diploma for genetics practice.

On July 15th, 2014, the Moroccan Minister for Health presented in a press release its intention to forbid the free sale of DNA tests by Internet means and for paternity test in particular. The lack of legal framework and the potential misuse of the test by a man who wants to avoid parental responsibilities based on a negative test were areas of concern. The Moroccan justice approved only two laboratories in the whole country for judicial DNA tests, one in Rabat and the other one in Casablanca. (Pauline Chambost, 2014). This is a step to ensure quality control with regards to genetic tests.

Historically, abortion was legal only when it constituted a necessary measure to protect the health of the mother (Dr Anwar Cherkaoui, 2001). The revision of Islamic prophetic texts about embryo creation, lead some scholars to say that it is not forbidden to have abortion before the first 4‐month is finished, but that after the completion of 4 months, abortion is forbidden. They rely on a prophetic text saying: “*Verily, each of you is brought together in his mother's abdomen for forty days in the form of a drop of fluid. Then it is a clinging object for a similar [period]. Thereafter, it is a lump looking like it has been chewed for a similar [period]. The angel is then sent to him and he breathes into him the spirit”*.

On June 9th, 2016 the Council of government adopted the new penal code which expands abortion rights, this penal law will be reviewed by Parliament room for admission. Abortion will be legal in three cases: when the woman is a victim of rape or incest after opening of a judicial inquiry, when she is affected by mental disorders (but the list of these disorders was not defined in the bill yet), and finally in case of fetal deformation. The national order of doctors will have to define the precise list of the deformations.

In the first two cases, the abortion has to take place within 90 days after the beginning of the pregnancy. In case of fetal malformation, the abortion can be made before the 120th day of pregnancy, even more when the disease needs more time to be delineated but without exceeding 22 weeks. In every case, the pregnancy termination has to be made by a doctor in a public hospital center or in an approved private Hospital (Reda Zaireg 2016) (Table 3).

**Table 3:** Abortion policy in the previous and the new penal code article

Abortion policy penal law article	Previous 453 penal law article	2016 new penal law article	Conditions
Grounds on which abortion is permitted	Necessary measure to protect the health of the mother	Necessary measure to protect the health of the mother	
		Pregnancy results from a Rape or crime of incest	‐ 3 days delay to think about a decision
			‐ Information about abortion risk
			‐ Information about judicial procedures if choice to keep pregnancy
			‐ Abortion have to be done in a public hospital or accredited clinic
			‐ Abortion have to be done before 90 days of pregnancy
		Mother with mental illness	‐ Husband consent
			‐ Parents consent if mother unmarried
			‐ Legal tutor consent
			‐ Legal institution consent
		Fetus with grave malformations	‐ Abortion before 120 days of pregnancy'
			‐ List of malformations not yet established
		Fetus with genetic conditions	‐ Abortion before 120 days of pregnancy
			‐ List of genetic conditions not yet established

## Pharmacogenomics, Personalized Medicine, Genetic Predisposition to Tuberculosis, and Lab Standardization

Pharmacogenomics is the collective effect of the genome on drug metabolism, related to personalized medicine, with the aim of defining for each patient the most appropriate treatment suitable for his genetic characteristics and biological particularities.

Morocco is an endemic country of tuberculosis. To develop new preventive and therapeutic strategies, it is important to understand the pathophysiology of the disease. Thus the identification of genetic factors predisposing to tuberculosis is fundamental. Among the factors that explain the large individual variability in responding to *Mycobacterium tuberculosis*, the role of the host genetic factors is elucidated. Through numerous association studies performed in Morocco, the interaction between host and bacterium in this disease opens the door to personalized medicine which can be extrapolated to other genetic conditions and personalized drug response (El Baghdadi et al. 2013).

It is imperative that laboratories provide high quality, reliable, and accurate testing. Today, Moroccan biologists have to be dynamic in driving their labs toward the international certification ISO9001, even toward the accreditation according to the standard ISO 15189.

Recently the Moroccan newsletter “Libération” in 2015 has published the news of ISO 9001 certification for Rabat National Institute of Hygiene operating many biological tests including genetic testing.

## Research in Genetics

Fes University of Al‐Qarawiyin built in 859, by Fatima Al‐Fihri, has the distinction of being the world's very first university. Yes, it was a Muslim woman who pioneered a model of higher learning coupled with the issuance of degrees of various levels. Pope Sylvester II graduated from the same university and was the first to introduce Arabic numerals to medieval Europe. One of the university's most famous students was a Jewish physician and philosopher, Maimonides (Joseph and Najmabadi 2003; Swartley 2005).

Research in Morocco is investigator‐driven without input from the government regarding research priorities. Research funding relies on international, formal or informal, scientific cooperation. The latter exists mainly with France (Mina Kleiche, 2002, 2002, 2002). Some diagnostic and research services offered to patients are sustained by small networks with international cooperation. Some Moroccan genetic departments are connected to Orphanet (European reference portal network for information on rare diseases and orphan drugs), or associated with INSERM (French National Institute of Health and Medical Research).

**Figure 2:**
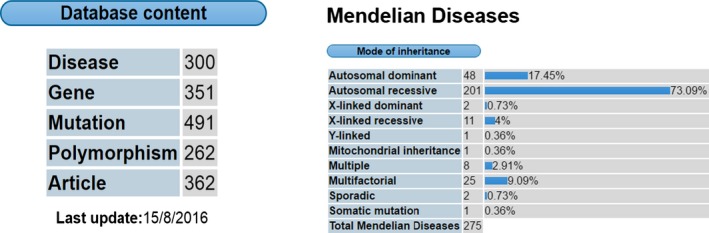
MGDD general statistics, disorders percentage according to the mode of inheritance (Institut Pasteur Maroc, 2016).

The Human Molecular Genetics Unit at Morocco Pasteur Institute can be used as an example of genetic research in Morocco. This unit focuses on molecular investigation of hereditary deafness, type 2 diabetes and the role of the Y chromosome in sex determination and male infertility. Knowing that Human mutation databases can be divided into general, locus‐specific, and national and ethnic mutation databases, this lab has the mission to collect and document mutations and frequencies of polymorphisms reported in Moroccan population (mainly from published articles available in Pub Med and other online resources). This data collection, the Moroccan Genetic Disease Database (MGDD,) is an open resource available for researchers and clinicians in an easy web‐based access: http://mgdd.pasteur.ma/ (Claustres et al. 2002; Stenson et al. 2003; Hamosh et al. 2005) (Figure 2 and Table 4).

**Table 4:** MGDD presentation of the highest mutations and polymorphisms rate attributed to some genetic condition (Charoute et al. 2014)

Diseases	Mutation	Polymorphism	Total
Susceptibility to type 2 diabetes mellitus (T2DM)	0	43	43
Susceptibility to familial breast ovarian cancer 2	10	22	32
Susceptibility to spermatogenic failure	0	29	29
Susceptibility to familial breast ovarian cancer 1	4	21	25
Ataxia‐telangiectasia	14	11	25
Beta thalassemia	18	0	18
Susceptibility to *Mycobacterium tuberculosis*	0	16	16
Phenylketonuria	14	0	14
Primary congenital A glaucoma 3	11	0	11
Retinoblastoma	11	0	11

The Mediterranean Founder Mutation Database (MFMD) is another example of Moroccan genetics initiative to offer web‐based access to founder mutation information of the country's Mediterranean population: http://mfmd.pasteur.ma/. This database contains 383 founder mutations found in 210 genes related to 219 diseases and provide an overview about the migration history of the Mediterranean population (Charoute et al. 2015).

To recapitulate, medical and genetics researchers in Morocco are in general struggling to conduct their research. Morocco, as part of the Arab world and according to the 2013 Scimago Institutions Rankings report, was ranked as having a weak output and of low scientific impact (El‐Azami‐El‐Idrissi et al. 2013; ScimagoLab, 2013).

Table 5 displays some genetics literature starting from 1988 with genetics linkage analysis, and continuing recently with some important publications in different genetics subdivisions and using new emerging genetic technologies. Enormous amounts of Moroccan genetics studies require international collaboration as NGS and Array CGH are still unavailable in the country (Table 5).

**Table 5:** Genetics studies and literature using PubMed scientific publications Browser (Sefiani et al. 1988; Tajir et al. 2012; Grant et al. 2013; Makrythanasis et al. 2014; Mansouri et al. 2014; Natiq et al. 2014; Qrafli et al. 2014, 2014; Janati Idrissi et al. 2015; Ratbi et al. 2015; Twigg et al. 2016; Elalaoui et al. 2016; Guaoua et al. 2016; Jouali et al. 2016)

Author	Title	Genetics method	Field	Year
Guaoua S et al.	NAT2 Genotypes in Moroccan Patients with Hepatotoxicity Due to Antituberculosis Drugs.	Sanger sequencing and real‐time polymerase chain reaction.	Pharmacogenetics	2016
Elalaoui SC et al.	Further evidence of POP1 mutations as the cause of anauxetic dysplasia.	Sanger sequencing	Mendelian genetics	2016
Jouali F et al.	First application of next‐generation sequencing in Moroccan breast/ovarian cancer families and report of a novel frameshift mutation of the BRCA1 gene.	NGS‐targeted sequencing	Cancer genetics	2016
Twigg SR et al.	Acromelic frontonasal dysostosis and ZSWIM6 mutation: phenotypic spectrum and mosaicism.	NGS and deep sequencing for mosaicism evaluation	Mendelien genetics	2016
Janati Idrissi M et al.	TPMT alleles in Moroccan.	Allele‐specific PCR and PCR‐RFLP genotyping	Pharmacogenetics	2015
Ratbi I et al.	Heimler Syndrome Is Caused by Hypomorphic Mutations in the Peroxisome‐Biogenesis Genes PEX1 and PEX6.	Exome sequencing	Metabolic genetics	2015
Mansouri M et al.	A novel nonsense mutation in SCN9A in a Moroccan child with congenital insensitivity to pain.	Sanger sequencing	Mendelian genetics	2014
Makrythanasis P et al.	Diagnostic exome sequencing to elucidate the genetic basis of likely recessive disorders in consanguineous families.	Exome sequencing with Array CGH	Mendelien genetics	2014
Natiq A et al.	A new case of de novo 19p13.2p13.12 deletion in a girl with overgrowth and severe developmental delay.	Cytogenetics, molecular cytogenetics, array CGH, Methyl PCR	Clinical Cytogenetics	2014
Qrafli M et al.	The CYP7A1 gene rs3808607 variant is associated with susceptibility of tuberculosis in Moroccan population.	Real Time PCR and RFLP‐PCR genotyping	Genetic association studies	2014
Grant AV et al.	Age‐dependent association between pulmonary tuberculosis and common TOX variants in the 8q12‐13 linkage region.	Positional‐cloning approach for linkage‐disequilibrium mapping of an identified susceptibility locus in chromosomal region.	Genetics Linkage analysis	2013
Tajir M et al.	Pyruvate dehydrogenase deficiency caused by a new mutation of PDHX gene in two Moroccan patients.	Sanger sequencing	Mitochondrial genetics	2012
Sefiani A et al.	Linkage studies do not confirm the cytogenetic location of incontinentia pigmenti on Xp11.	X chromosome DNA probes	Genetic linkage analysis	1988

Historically, Moroccans were pioneers in several scientific domains. To promote medical research in Morocco, serious efforts and several strategic goals must be agreed on by all stakeholders, scientists, and decision makers. The strategy should include upgrading research infrastructure and equipment, providing sufficient funds and high‐quality training, as well as promoting excellence. Additionally, scientists working abroad should be seen as an asset. A medical research council – inspired from the US National Institutes of Health, the Medical Research Council in the UK, and INSERM in France – is necessary to establish strategies that promote medical and health research in collaboration with international institutions (El‐Azami‐El‐Idrissi et al., 2013; ScimagoLab. 2013).

Recently, in 2016, new research genetic centers are emerging either by the endorsement of the kingdom's highest authority or by private institutions. Three examples are highlighted:

- Princess Lalla Salma, chairwoman of the Lalla Salma Foundation for Cancer Prevention and Treatment, inaugurated the First Cancer Research Institute in Africa. This institute was created as part of a public interest group represented by Fes Hassan II University hospital center, Fes Sidi Mohammed Ben Abdallah University and Lalla Salma Foundation (Foundation Lalla Salma, 2016)
- A partnership between Rabat Abulcasis Health Sciences International University (UIASS) and Paris (Imagine) Institute of genetic diseases, the main genetic research institute in France. The objective aims to better understand genetic diseases, bringing diagnostic, and therapeutic solutions to Moroccan patients and their families (Doctinews, 2016)
- A laboratory of genetic engineering will be created at Fes Euro‐méditerranéenne University. A framework agreement was signed between Dassault systems and the ministry of the Higher education. This agreement has the objective of creating treatments for diseases from which the African population suffers (Doctinews, 2016)

## Conclusion

Genetics is a rapidly evolving field relying on technological advances and with a direct effect on society. Nevertheless, in Morocco, it is difficult to establish – if impossible outside very specialized departments – a diagnosis for many genetic conditions and to obtain exact data on prevalence of these diseases. According to international projections, the number of newborns who are or will be affected by genetic diseases is considered to be 5% in Morocco. Morocco needs to invest in genetics research, diagnosis of genetic disorders and clinical genetics services, including genetic counseling to help its citizens who are living with or at risk for genetic disorders. As Marc Lévy stated, “*The loss of a child is a wound which never heals*;* La perte d'un enfant est une plaie qui ne cicatrise jamais;* فقدان طفل جرح لا يندمل”
